# Optical sensor using space-domain active fiber cavity ringdown technique

**DOI:** 10.1038/s41598-022-17565-6

**Published:** 2022-08-04

**Authors:** Wenjia Chen, Yiwen Ou, Chunfu Cheng, Yuanchang Zhu, Wen Xiao, Hui Lv

**Affiliations:** 1 Hubei Engineering Technology Research Center of Energy Photoelectric Device and System, Hubei University of Technology, Wuhan, 430068 China; 2 School of Science, Hubei University of Technology, Wuhan, 430068 China

**Keywords:** Optics and photonics, Physics

## Abstract

A novel active fiber cavity ringdown (FCRD) technique using frequency-shifted interferometry (FSI) is proposed for the first time. Using this scheme, external parameters can be monitored in the space domain by measuring the ringdown distance instead of ringdown time. A bidirectional erbium-doped fiber amplifier (Bi-EDFA) is employed to compensate the inherent cavity loss for achieving higher sensitivity. And two band-pass filters are used to reduce the amplified spontaneous emission (ASE) noise of the Bi-EDFA. Compared with the well-known time-domain active FCRD scheme, our proposed method enables us to avoid using pulsed laser needed in time-domain active FCRD, it uses continuous-wave laser to inject into the fiber cavity and stabilize the optical power in the fiber cavity, which can suppress the baseline drift of ringdown signal caused by the gain fluctuations of the EDFA and thus improve the detecting precision. Moreover, this novel method enables us to use differential detection method for further reducing the ASE noise, and thus eliminating the baseline drift of ringdown signal. A magnetic field sensor was developed as a proof-of-concept demonstration. The experimental results demonstrate that the proposed sensor with a sensitivity of 0.01537 (1/km·Gs) was achieved. This is the highest magnetic field sensitivity compared to the time-domain active FLRD method. Due to the reduced ASE noise, the stability of the proposed sensing system was also greatly improved.

## Introduction

Fiber cavity ringdown (FCRD) sensing technique is a highly sensitive method for measuring optical losses^1–3^. Similar to conventional CRD scheme, the cavity loss can be determined from the decay rate usually called ringdown time of the pulsed laser. But unlike conventional CRD method, in which the light bounces back and forth between two mirrors, FCRD usually uses a pair of fiber directional couplers with high splitting ratio to form the fiber cavity for achieving the multi-pass approach. Compared to mirror-based cavity, a fiber cavity has the advantages of being alignment-free, robustness, low cost and suitable for large-scale, multi-function sensor network, which has made FCRD becoming a popular choice for many applications such as gas^4^, liquid^5,6^, refractive index^7^, strain^8^, temperature^9^, magnetic field sensing^10^ and so on. However, fiber cavity has its shortcoming of the large inherent cavity loss due to the large insertion loss of fiber couplers and sensor heads, which leads to a poor sensitivity.

In order to improve the sensitivity, one simple way to achieve this goal is to reduce the insertion losses of the sensor heads, but the improvement is still limited. Another approach is to compensate the inherent cavity loss by introducing an erbium-doped fiber amplifier (EDFA) to the fiber cavity. As the EDFA is served as the gain source, this new FCRD is usually named as time-domain active FCRD or amplified FCRD^11–21^. In 2001, the active FCRD technique was firstly proposed by George Stewart and the sensitivity was improved because the inherent cavity loss can be sufficiently compensated by the EDFA^11^. However, the active FCRD sensing method also brings two new problems. One is the gain fluctuation of the EDFA, which results in the nonexponential decay of the ringdown signal and thus degrades the measurement precision and long-term stability^20,21^. Another is the amplified spontaneous emission (ASE) noise produced by EDFA, which causes the baseline drift of ringdown signal and reduces the stability of the sensing system^12,13,17^. To minimize the impact of gain fluctuation, a gain clamped EDFA was used in the fiber cavity to reduce the gain fluctuation effect^14,16^, but the gain fluctuation still exists because the pulsed laser was used in time-domain active FCRD to excite the fiber cavity and thus it cannot provide the power stabilization in the fiber cavity, so the stability was usually only about 10% which was not suitable for the practical application^18^. Fortunately, a chaotic laser was proposed to stabilize the laser power in the fiber cavity and the influence of the gain fluctuation was effectively suppressed, therefore, a good stability of 2.84% was achieved recently^19^. In order to improve the stability, an adaptive filter was suggested to suppress the ASE noise^12,13^, however, it is impossible to completely eliminate it and thus the stability was still not sufficient.

Conventional FCRD method belongs to time-domain sensing technique because it usually uses pulsed laser to excite the fiber cavity and measures its decay rate (ringdown time) in the time domain. As this scheme is based on using an expensive pulsed laser, a fast detector and a fast data acquisition card (DAQ), it incurs high instrument costs and limits its applications. To solve the above issues raised by conventional FCRD scheme, a space-domain passive FCRD technique was proposed based on frequency-shifted interferometry (FSI), which usually called as FSI-FCRD^22,23^. This novel scheme does not rely on any time-domain measurement of a decaying pulsed laser. Instead, it is able to deduce the cavity loss information by measuring the ringdown distance. Compared with the conventional FCRD method, this new scheme was more cost-effective because it utilizes a low-cost continuous wave (CW) laser and slow detector without the need of expensive pulsed laser and fast electronics. Due to its excellent characteristics, such as low cost and high stability, the space-domain passive FCRD technique has attracted more and more interests and been widely used for many sensing applications, such as strain sensing^25^, refractive index detection^24^, magnetic field sensing^27^ and so on. However, it has the same problem of large inherent cavity loss as the time-domain passive FCRD sensing method, which limits the sensitivity of the sensing system. To meet the requirements of many practical applications for high sensitivity, a space-domain active FCRD is urgently required to develop. However, to the best of our knowledge, it has not yet been reported.

In this paper, we proposed the space-domain active FCRD technique for the first time and successfully carried out proof-of-concept experiments to measure magnetic field. The experimental results show that this novel scheme can not only significantly increase the sensitivity, but also it is able to improve the stability of sensing system due to the reduced baseline drift of ringdown signal caused by the gain fluctuation by using a CW source to stabilize the laser power in the fiber cavity. It is fundamentally different from the time-domain active FCRD technique since the latter requires pulsed laser to inject the fiber cavity and thus a large gain fluctuation is generated. This paper is organized as below. In “Principle”, we introduce the principle of the space-domain active FCRD. In “Experimental setup” and “Results and discussion” we respectively show the experimental setup and experimental results of the space-domain active FCRD magnetic field sensing system. The last section is a short summary.

## Principle

**Figure 1:**
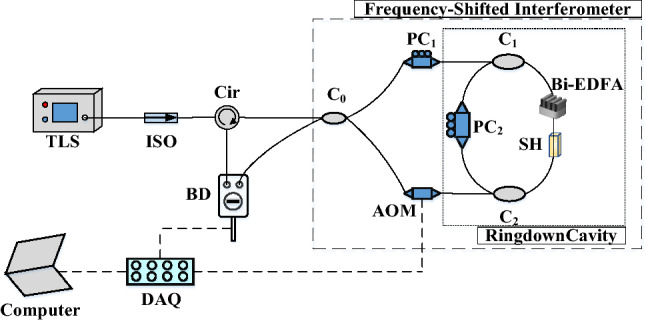
Schematic diagram of the space-domain active FCRD sensing system. *TSL* tunable semiconductor laser; *ISO* isolator, *Cir* circulator, *C*_*0*_ 3 dB fiber coupler, *C*_*1*_* and C*_*2*_ high splitting-ratio fiber couplers, *PC*_*1*_* and PC*_*2*_ polarization controllers, *SH* sensor head, *Bi-EDFA* bidirectional erbium-doped fiber amplifier, *AOM* acousto-optic modulator, *BD* balanced detector, *DAQ* data acquisition card.

The schematic diagram of the space-domain active FCRD sensing system is depicted in Fig. 1. From Fig. 1 we can see that a Sagnac interference loop, with an asymmetrically-embedded frequency shifter, constitutes a frequency-shifted interferometer^28^. An active fiber cavity is composed by two fiber couplers (C_1_ and C_2_) with high splitting ratios, a sensor head (SH), a bidirectional erbium-doped fiber amplifier (Bi-EDFA), which is inserted into the frequency-shifted interferometer to form the space-domain active FCRD sensing system. The working principle of space-domain active FCRD sensing system can be described as follows. The CW light generated from the tunable semiconductor laser (TSL) is coupled into the frequency-shifted interferometer through the circulator (Cir) and the fiber coupler C_0_. It produces two light beams circulating the fiber cavity in counter-propagating directions. A Bi-EDFA as shown in Fig. 1 is incorporated into the fiber cavity to amplify the intensity of the two light beams in the cavity. Consequently, it compensates the inherent cavity loss, the light-sample interaction time is prolonged, and the two light beams attenuate more slowly. Small amounts of the light beams leak out from the fiber cavity and encounter in the coupler C_0_, after completing each roundtrip in the cavity. The interference occurs between the two counter-propagating light beams exiting from the fiber cavity after going through the same number of roundtrips. If a frequency range of *Δf* is swept from an initial frequency *f*_0_ over a duration of *t*_*sw*_, the differential interference signal at the balanced detector (BD) will be a sinusoidal function of time *t*^22,23^

1 $$\begin{gathered} \Delta I \propto \sum\limits_{m = 0}^{\infty } {I_{m} \cos \left[ {2\pi \frac{{n\left( {mL + L_{0} } \right)}}{c}f} \right]} \hfill \\ { = }I_{m} \cos \left[ {2\pi \frac{{n\left( {mL + L_{0} } \right)}}{c}(\frac{\Delta f}{{t_{sw} }}t + f_{0} )} \right] \hfill \\ \;\;\; = I_{m} \cos \left[ {2\pi \cdot F_{m} \cdot t + \varphi_{0m} } \right] \hfill \\ \end{gathered}$$

where *n* is the refractive index of the single-mode fiber, *L* is the length of the fiber cavity, *L*_0_ is a constant, *f* is the frequency shift generated by the frequency shifter, *c* is the propagation speed of the light in vacuum, *φ*_0*m*_ is a fixed initial phase without consequence to our calculation, *F*_m_ = *n*(*mL* + *L*_*0*_)*Δf*/(*ct*_*sw*_) (m = 0, 1, 2, …) is the oscillation frequency that has a linear relationship with the roundtrip number *m*^22^. As *c* is very large, the sensing system can work under the low oscillation frequency *F*_*m*_, that is to say, slow detection is achieved using FSI and thus the cost is reduced*. I*_m_ is the intensity of the interference light after *m* roundtrips:

2 $$I_{m} = I_{0} \cdot \exp ( - m\alpha_{0} ) = I_{0} \cdot \exp ( - \frac{l}{L}\alpha_{0} )$$

where *I*_*0*_ is the initial intensity, *l* = *mL* is the distance traveled by the light in the fiber cavity, and $\alpha_{0}$ is the net cavity loss, which contains the total fiber transmission loss $\alpha_{c}$ and the gain *G* of the Bi-EDFA in the fiber cavity, and can be given by:

3 $$\alpha_{0} { = } \, \alpha_{c} - G{ = }\alpha_{AR} { + }\alpha_{ST} { + }\alpha_{FS} { + }\alpha_{IR} - G$$

where $\alpha_{AR}$ represents the fiber absorption loss, $\alpha_{ST}$ is the fiber scattering loss, $\alpha_{FS}$ is the fiber fusion loss, and $\alpha_{IR}$ is the insertion loss of the components including the two fiber couplers, the polarization controller and the sensor head (SH). $\alpha_{c} { = }\alpha_{AR} { + }\alpha_{IR} { + }\alpha_{ST} + \alpha_{FS}$ represents the inherent cavity loss, which is larger than the net cavity loss $\alpha_{0}$.

As it can be seen from Eqs. (1)–(3), *I*_*m*_ is an exponentially decay function of the propagation distance *l*, while *F*_*m*_ is a linear function of *l* according to the relation *l* = *mL.* Therefore, by linearly sweeping the frequency shift *f* and applying fast Fourier transform (FFT) to Δ*I*, one can obtain the Fourier spectrum of Δ*I* which display a series of exponentially decaying peaks located at *F*_*m*_. After converting the frequency *F*_*m*_ to the propagation distance *l*^*’*^ using the formula *l*^*’*^ = *l* + *L*_*0*_ = *cF*_*m*_*/n*, the Fourier spectrum of Δ*I* will become a ringdown transient as the propagation distance^22,23^, which indicates our proposed method belongs to a space-domain FCRD technique realized by using FSI scheme. The distance required for the light intensity to decrease to 1/e of the initial light intensity can be defined as the ringdown distance, which is analogous to the ringdown time^1^. When no external action applied on the SH, the ringdown distance $\Lambda_{0}$ can be written as:

4 $$\Lambda_{0} = \frac{L}{{\alpha_{0} }}$$

When an external action *P* (magnetic field, pressure, strain, etc.) is applied on the SH, an additional loss $\alpha_{s}$ occurs, which results in a change in the ringdown distance. In this case, the ringdown distance $\Lambda$ can be rewritten as:

5 $$\Lambda = \frac{L}{{\alpha_{c} + \alpha_{s} - G}}{ = }\frac{L}{{\alpha_{c} + \xi l_{s} P - G}}$$

where $\alpha_{s} = \xi l_{s} P$, $\xi$ is the external-action-induced absorption coefficient^26^, and $l_{s}$ is the length of the SH. Combining Eq. (4) with Eq. (5), we have:

6 $$(\frac{1}{\Lambda } - \frac{1}{{\Lambda_{0} }}){ = }\frac{{\alpha_{s} }}{L}{ = }\frac{{\xi l_{s} }}{L}P{ = }kP$$

Obviously, the external parameter is determined by measurement of ringdown distances $\Lambda$ (with the external action applied) and $\Lambda_{0}$(without the external action applied), and the reciprocal difference ($1/\Lambda - 1/\Lambda_{0}$) of ringdown distance changes linearly with the change of the external action, for a given space-domain active FCRD sensor. The slope $k{ = }\xi l_{s} /L$ represents the sensor’s sensitivity to the sensing activity, which can be tailored by adjusting the length of SH and the cavity length. Additionally, the detection limit is also an important indicator of system performance as well, which is defined as the minimum detectable external parameter *P*_*min*_. It can be obtained by taking the derivation of both sides of Eq. (6)

7 $$P_{\min } = \frac{1}{{kN_{e} L}} \cdot \frac{\delta \Lambda }{{\overline{\Lambda }}}$$

where $N_{e} = \Lambda_{0} /L$ is the effective number of roundtrips traveled by the light within the ringdown distance $\Lambda_{0}$, which essentially represents the multiple rounds of interaction in the SH. Therefore, compared with the detection limit of an intensity-based fiber optic sensor, our proposed sensor will improve the detection limit by a factor of *N*_*e*_. $\delta \Lambda$ and $\overline{\Lambda }$ denote the standard deviation and the averaged value of the ringdown distance under the condition of no external action applied on the SH, respectively. The ratio $\delta \Lambda /\overline{\Lambda }$ can be defined as the stability of the proposed sensing system, which is similar to the definition of stability in the time-domain FCRD sensing system^1^. Equation (7) indicates that the high detection limit can be expected under the good stability, which is very important to the practical application. As the gain of Bi-EDFA can decrease the cavity loss and prolong the ringdown distance, the space-domain active FCRD technique will have larger effective circulating number and thus results in a higher sensitivity and detection limit. However, a higher sensitivity usually results in a smaller dynamic range, and vice versa^29^. That is to say, they should be compromised according to the requirements of the practical applications.

## Experimental setup

**Figure 2:**
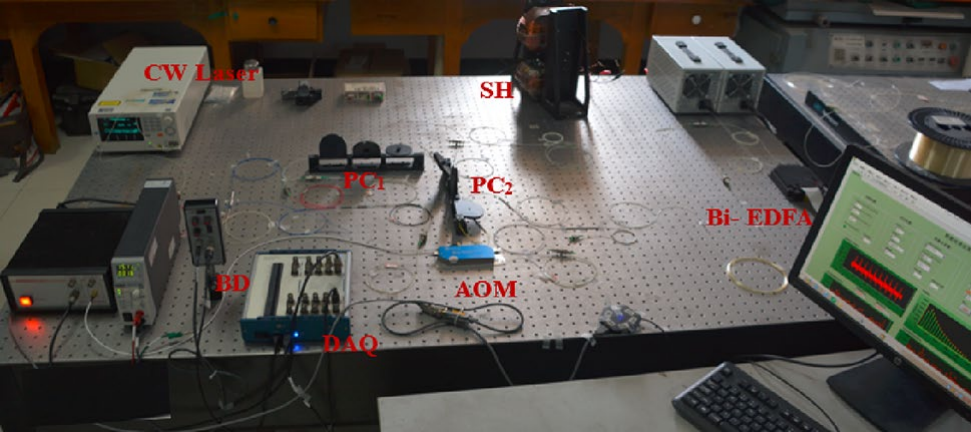
The experimental setup of the space-domain active FCRD magnetic field sensing system.

In order to verify the feasibility of our proposed concept, a space-domain active FCRD sensing system was constructed for magnetic field measurement as shown in Fig. 2. A tunable semiconductor laser (TSL, Santec TSL-550) was employed to generate CW light. During the magnetic field sensing experiments, the output power of TSL was set to 9 mW at 1530.37 nm. An acousto-optic modulator (AOM, Brimrose, AMM-100-20-25-1550-2FP) served as the frequency shifter, and its sweeping frequency was set to rise from 90 to 110 MHz with steps of 0.02 MHz and frequency scan periods of 1 s. Two fiber couplers with a splitting ratio of 99.5:0.5 constituted the fiber cavity with a length of ~ 130 m. Two polarization controllers (PC_1_ and PC_2_) were used to optimize the polarization state of the CW light for obtaining the visible interference fringe. The differential interference signal was detected by the balanced detector (BD, New Focus Model 2117), and then recorded by a data acquisition card (DAQ, NI USB-6361) at a sampling rate of 100 kS/s. Note that this detection speed is much slower than that of conventional time-domain FCRD sensing system^1,16^. The collected signal was then transmitted to the desktop computer and processed by a Labview program in real time.

**Figure 3:**
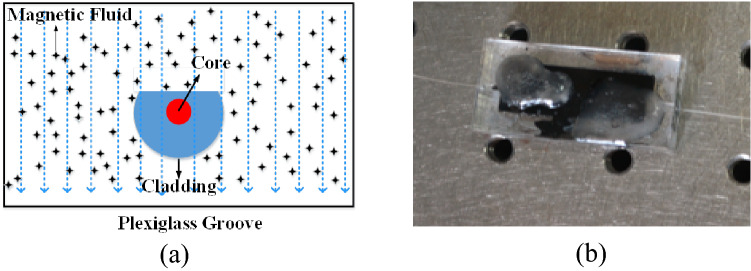
**(a)** Schematic diagram of magnetic field sensor head; **(b)** physical view of the magnetic field sensor head.

As a proof-of-principle demonstration, we introduced the cavity loss induced by magnetic field, and a side-polished fiber (SPF) coated with magnetic fluid (MF) was utilized as the SH (see Fig. 3a,b) for magnetic field sensing. The SPF (Micro photons Technology Co., NIR-SPF-W1550-2) was fabricated by side polishing the cladding of a single-mode fiber to 5–6 μm from the core, which significantly enhanced the evanescent field effect in the polished area with a length of 17 mm. Compared with the tapered fibers, the SPF has stronger mechanical strength because the other side of the fiber is intact. Moreover, the fabrication method requires no pigtailing and no intrusion into the core of the fiber, which produces a robust structure. Then the SPF was encapsulated in a plexiglass box filled with the MF (Water-based Ferrofluid, EMG-603P, Ferrotec (USA) Corp.). The MF was a stable water-based ferrofluid with Fe_3_O_4_ as the magnetic nanoparticles. Due to the agglomeration of Fe_3_O_4_ particles and formation of a long-chain structure that is parallel to the magnetic field direction^28^, the refractive index and absorption coefficient of MF will change with the change of the ambient magnetic field strength. When the magnetic field was applied on the proposed SH, a magnetic-field-induced cavity loss will emerge, which results in the change of the ringdown distance. Consequently, the applied magnetic field can be measured by monitoring the change in ringdown distance. The two small holes on the top of the plexiglass box was packed with paraffin to prevent the MF from overrunning the box. A self-made magnetic field generating device created the magnetic field in the experiments, which was composed by two square iron cores wound tightly with copper wire and fed by a digital regulated direct-current (DC) power supply. The direction of the magnetic field was perpendicular to the propagation direction of the CW light in the fiber cavity. A gaussmeter (Pafei Hangzhou, BST-801) was employed to measure the magnetic field as a benchmark.

It is worth noting that the EMG-603P MF used here was not diluted using distilled water as in our recent work^31^. The space-domain ringdown event could not be observed anyway before connecting the Bi-EDFA inside the fiber cavity, which indicates that high concentration of MF causes a large cavity loss. However, after a customized Bi-EDFA (Tianjin Junfeng Technology Co., EDFA-WDM-C-MB) was utilized to realize the bi-directional amplification of the CW light intensity, the space-domain ringdown transient could be observed immediately. It experimentally reveals that the Bi-EDFA can compensate the cavity loss and prolong the ringdown distance. Two band pass filters were encapsulated in the Bi-EDFA to filter out the ASE noise, each with a central operating wavelength of 1,530.33 nm and a 3-dB bandwidth of 0.8 nm. The pump current of the Bi-EDFA were set to 135 mA during the experiments.

## Results and discussion

**Figure 4:**
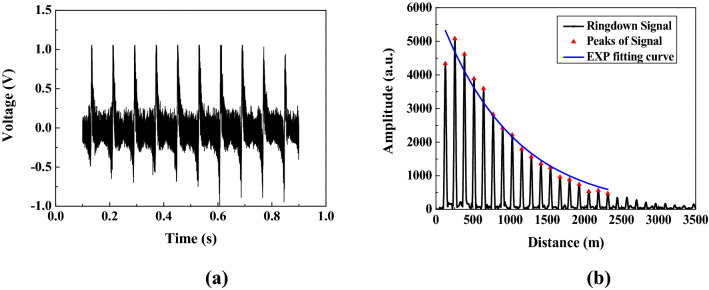
The typical signals measured by the space-domain active FCRD technique. **(a)** The time-domain differential interference signal; **(b)** the corresponding ringdown transient in the space domain.

A typical differential interference signal is shown in Fig. 4a when there was no external magnetic field was applied on the SH, and the corresponding ringdown waveform in the space domain (see Fig. 4b) was obtained after taking FFT of the differential interference signal. The peaks on the ringdown signal can be extracted by applying a peak extraction algorithm. By fitting these peaks with an exponential (EXP) function, an exponential decay curve was obtained. Then the ringdown distance was calculated out to be 1,028 m according to its definition, and cavity length was ~ 129 m by calculating the average space interval between two adjacent peaks. According to Eq. (4) the net cavity loss can be deduced to be 0.548 dB in the absence of an external magnetic field.

**Figure 5:**
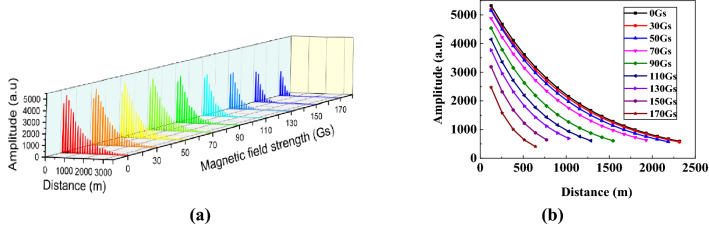
The typical signals measured by the space-domain active FCRD technique. **(a)** the time-domain differential interference signal; **(b)** the corresponding ringdown transient in the space domain.

**Figure 6:**
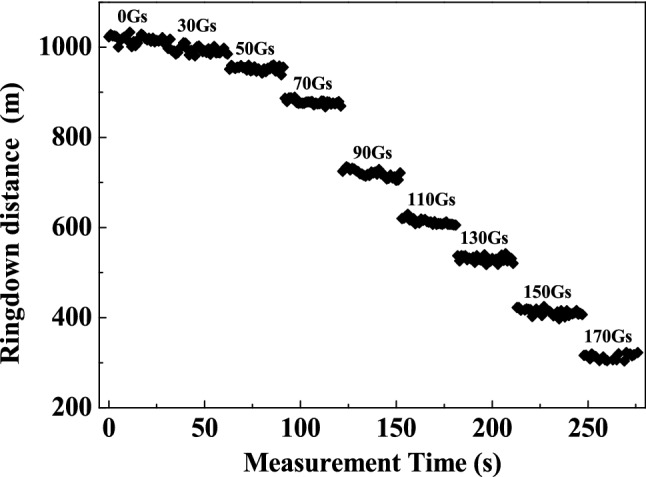
Ringdown distance versus magnetic field strength.

**Figure 7:**
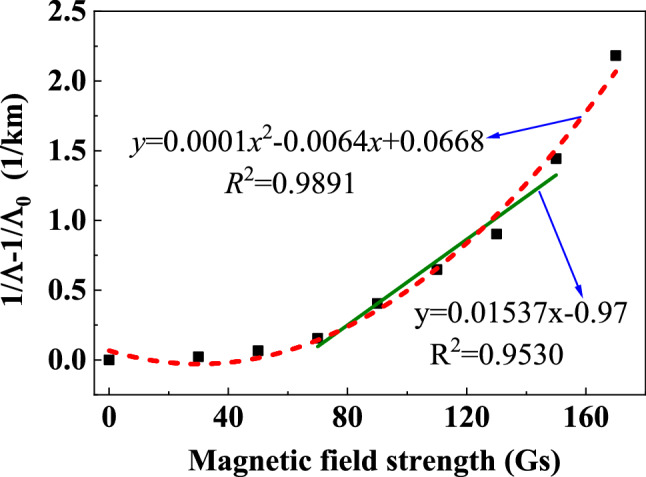
Reciprocal difference of ringdown distance.

To characterize the response of the proposed sensing system to magnetic field and evaluate its sensitivity, the magnetic fields ranged from 0 to 170 Gs were applied on the SH, and the ringdown signals were measured. The obtained results are illustrated in Fig. 5a. By using peak extraction algorithm and EXP fitting algorithm, the obtained exponential decay curves under different magnetic field strengths are depicted on Fig. 5b. As the magnetic field strength rises, the attenuation of the ringdown signal becomes faster, which shows the ringdown distance decreases with the increase of the magnetic field strength. This is because: when there is no magnetic field applied, the magnetic nanoparticles are uniformly distributed in the carrier liquid; whereas when the magnetic field is applied, the magnetic nanoparticles gradually cluster along the direction of the magnetic field. As the intensity of the magnetic field increases, more and more magnetic nanoparticles are arranged into short-chain and long-chain structures, causing the increase in the refractive index of the MF^30^. As a result, more evanescent wave leaks out from the SPF, the cavity loss increases, and thus the ringdown distance decreases. This result can also be clearly observed in Fig. 6, in which the measurement was repeated 30 times under each magnetic field strength to reduce the measurement errors. By calculation, the average ringdown distance measured by the sensing system reduced from 1017 m to 313 m. To clearly demonstrate the response characteristics of our proposed sensing system, the relationship between the reciprocal difference of ringdown distance (1*/*Ʌ − 1*/*Ʌ_0_) and the magnetic field strength *H* was plotted as shown in Fig. 7. As the magnetic field strength increases, the reciprocal difference of ringdown distance gradually increases. Through linear fitting, it can be found that, in the range of 70–150 Gs of magnetic field intensity, the linear fitting degree R-squared reaches 0.9530. It indicates that the sensing system has a good linear response, which also agrees well with the theoretic result described in Eq. (6). Other regions show a non-linear change trend, which mainly caused by the initial magnetization of magnetic fluid^10^, and when the applied magnetic field strength exceeded 150 Gs, excessive cavity loss induced by magnetic field resulted in an unfaithful measurement and even the disappearance of the ringdown signal. The polynomial fitting was applied to all data points, and a nonlinear curve was obtained with a good R-squared of 0.9891. It indicates that the measurement range of the proposed scheme can be equal to or larger than 170 Gs. Although the linear response range is limited, a highly sensitivity would be expected as a smaller measurement range usually results in a higher sensitivity. The fitted slope is 0.01537, which represents that the sensitivity of the sensing system is 1.537 × 10^–2^ (1/km·Gs). By converting ringdown time/distance change as a function of magnetic field strength.

into cavity loss change for keeping units the same, it can be found that the sensitivities of the time-domain passive FCRD magnetic field sensors reported in^29^, the time-domain active FCRD magnetic field sensing system^30^, and the space-domain passive FCRD magnetic field sensing system^27^ were all lower than the sensitivity of our proposed sensing system as shown in Table 1. Obviously, the sensitivity of the space-domain active sensing system can be further enhanced by increasing the gain of Bi-EDFA, but large gain will bring a higher ASE noise, which will deteriorate the stability of the sensing system. In addition, according to the Eq. (6) the sensitivity also can be further improved by increasing the length of the SH, but the measurement range will be compromised. Therefore, the sensitivity and measurement range of the proposed sensing system should be tailored according to the specific performance requirements of the practical applications.

**Table 1:** Comparison with conventional FCRD techniques.

Ref	Technique	Sensor head configuration	Sensitivity
^10^	Time-domain passive FCRD	Etched fiber taper coated with MF	8.07 × 10^–4^ dB/Gs
^30^	Time-domain active FCRD	U-bent SMF coated with MF	3.45 × 10^–3^ dB/Gs
^27^	Space-domain passive FCRD	Etched fiber taper coated with MF	1.05 × 10^–3^ dB/Gs
Our work	Space-domain active FCRD	Side-polished fiber coated with MF	8.59 × 10^–3^ dB/Gs

**Figure 8:**
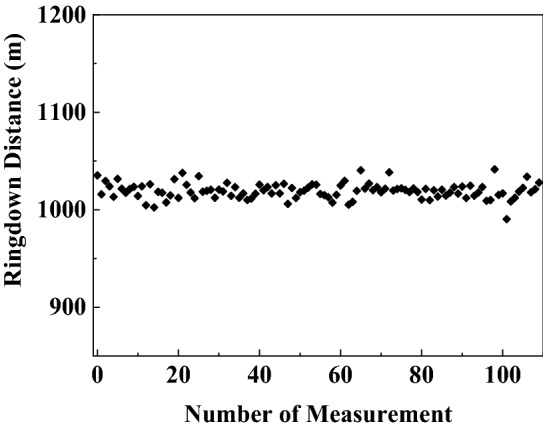
Stability measurement results of the proposed sensing system.

Finally, the stability of the sensing system was also tested. The ringdown distance was repeatedly measured one hundred times when no magnetic field was applied on the SH, and the results were demonstrated in Fig. 8. The obtained mean ringdown distance was 1019 m, and the standard deviation was 8.1 m. According to the definition of the stability in “Principle”, the stability of 0.79% was obtained, which is much better than that of the time-domain active FCRD sensing system with stability around 10%^1,13,18^ and also better than that of chaotic correlation FCRD sensing system with stability of 0.846% by using chaotic laser instead of pulsed laser to effectively suppress the gain fluctuations of EDFA^19^. The better stability of the proposed sensor system is attributed by the use of a CW light source to ensure the power stability in the fiber cavity for suppressing the baseline drift of ringdown signal caused by the gain fluctuations of the Bi-EDFA, the usage of differential detection to eliminate DC noise, and the essence of common-path interference to cancel common-mode noise. According to Eq. (7), the calculated detection limit of the proposed sensing system was 0.504 Gs, which is better than that of the FCRD-based magnetic field sensor reported in^31–33^. In addition, the effective number of roundtrips N_e_ was calculated to be about 8, which means 8 rounds of interaction in the SH. In other words, the detection limit is about eightfold enhancement than those of the conventional intensity-based sensors.

## Conclusions

In this paper, a space-domain active FCRD sensing technique by introducing the Bi-EDFA to compensate the inherent cavity loss was firstly proposed, and magnetic field sensing experiments were conducted to verify the concept. Compared to the conventional time-domain active FCRD scheme, this novel method does not need a pulsed laser and a fast detector, which reduces the system cost. It uses a CW laser to stabilize the laser power in the fiber cavity, and thus effectively suppress the baseline drift of ringdown signal caused by the gain fluctuations of the Bi-EDFA, offering a good stability for the sensing system. It also utilizes the different detection method to obtain high signal-to-noise ratio, and hence the stability of sensing system is further improved. In the magnetic field sensing experiments, we have achieved a highly sensitivity of 1.537 × 10^–2^ (1/km·Gs) and a good stability of 0.79%, both of which are better than those of time-domain active FCRD magnetic field sensing systems. The results show that the proposed method is cost-effective and suitable for many practical applications where high sensitivity and good stability are required.
